# Nano-Amounts of Glucagon Premixed With Fast-Acting Insulin Lispro: Effect on Insulin Absorption in Pigs

**DOI:** 10.1016/j.curtheres.2025.100803

**Published:** 2025-06-24

**Authors:** Ilze Dirnena-Fusini, Misbah Riaz, Sverre Christian Christiansen, Sven Magnus Carlsen

**Affiliations:** 1 Department of Clinical and Molecular Medicine, Faculty of Medicine and Health Sciences, Norwegian University of Science and Technology, Trondheim, Norway; 2 Department of Endocrinology, St. Olav’s Hospital, Trondheim University Hospital, Trondheim, Norway

**Keywords:** Animal experiments, Glucagon, Insulin, Insulin infusion systems

## Abstract

**Background:**

Glucagon leads to substantial but short-lived subcutaneous vasodilation. Using micro-amounts of glucagon at the insulin injection site increases insulin absorption.

**Objective:**

We hypothesized that a premixed solution of insulin and nanogram doses of glucagon would improve the pharmacokinetic and pharmacodynamic properties of subcutaneously injected insulin.

**Methods:**

In this series of proof-of-concept experiments, 17 anesthetized pigs were included. Nine pigs were included in the control groups; they received a subcutaneous injection of 10 U of insulin lispro (Lyumjev^Ⓡ^ or Humalog^Ⓡ^). Eight pigs were included in the glucagon groups; they received 10 U of a premixed insulin (Lyumjev^Ⓡ^ or Humalog^Ⓡ^)/glucagon solution (5 ng glucagon/unit of insulin). Arterial blood was frequently sampled for 210 minutes to assess insulin and glucose concentrations. The impact on glucose metabolism was evaluated through euglycemic clamp investigation.

**Results:**

When premixed insulin Lyumjev^Ⓡ^/glucagon was injected, insulin T_max_ decreased from 33 to 20 minutes (SE = 6.6, *P* = 0.08), and C_max_ was 2-fold higher than that in the control group (100 mU/L vs 46 mU/L, SE = 4.8, *P* = 0.007). When premixed insulin Humalog^Ⓡ^/glucagon was injected, T_max_ and C_max_ did not change significantly (*P* = 0.53 and *P* = 0.83, respectively). Insulin AUC for the first 15 minutes increased two-fold when insulin Lyumjev^Ⓡ^/glucagon was injected (946 mU×min/L vs 337 mU×min/L, SE = 196, *P* = 0.02). A similar trend was observed when Humalog^Ⓡ^/glucagon was injected (306 mU×min/L vs 65 mU×min/L, SE = 125), although this difference did not reach statistical significance (*P* = 0.102) compared with the control groups.

**Conclusions:**

This series of proof-of-concept experiments in anesthetized pigs indicate that premixing nanogram doses of glucagon in fast-acting insulin lispro formulations may speed up the absorption of subcutaneously injected insulin.

## Introduction

The primary obstacle to obtaining good glucose control for patients with type 1 diabetes (T1D) is the delayed subcutaneous absorption of even the most fast-acting insulins. The postprandial glucose excursions in patients with T1D are inadequately inhibited by this delayed insulin absorption and its corresponding effects on whole body glucose utilization, as many premeal boluses are injected shortly before or during a meal.^1^^,^^2^ People who are treated with multiple daily insulin injections or insulin pumps are recommended to inject the meal bolus of insulin 10 to 20 minutes before eating to minimize the effect of delayed insulin absorption. Despite these precautions, many individuals continue to experience excessive postprandial glucose excursions and suboptimal glucose control. Additionally, there is an increased incidence and severity of late postprandial hypoglycemia when more aggressive insulin treatment is used to reduce postprandial hyperglycemia.

After decades of research and development by pharmaceutical companies, various rapid-acting insulin formulations have been introduced in the market. The most rapid-acting is Lyumjev^Ⓡ^ (insulin lispro*), a fast-acting insulin formulation enhanced with citrate to increase capillary permeability and treprostinil to promote vasodilation, both of which accelerate insulin absorption. Nevertheless, even with these additives, it takes 40 to 45 minutes to reach insulin C_max_, and maximum effect on whole body glucose utilization is observed between 90 and 120 minutes.^3^ Another insulin lispro formulation is Humalog^Ⓡ^†; this formulation does not contain citrate or treprostinil and takes 30 to 90 minutes after bolus to reach insulin C_max_ (Eli Lilly and company data).

We previously reported a significant increase in local subcutaneous blood flow in healthy individuals after subcutaneous administration of 0.1 and 0.01 mg of glucagon.^4^ Additionally, in anesthetized pigs, we also observed that injecting 0.1 mg and 0.01 mg glucagon just before the insulin injections at the same subcutaneous site increased insulin lispro (Lyumjev^Ⓡ^) absorption, with the smaller glucagon dose enhancing absorption just as much as the higher dose.^5^ We also have unpublished data that shows that as little as 50 ng glucagon exert the same effect.

Simultaneously, we found in experiments on ourselves that even a 5.0 ng glucagon injection increased subcutaneous vasodilation significantly more than an injection of the same volume of saline (no ethical approval, data not published). Furthermore, injections of premixed solutions of insulin (Humalog^Ⓡ^, Lyumjev^Ⓡ^, NovoRapid^Ⓡ^, and FIASP^Ⓡ^ mixed with as little as 5.5 ng glucagon per unit of insulin) increase subcutaneous vasodilation 2 to 3 times more than an insulin-only injections (no ethical approval, data not published).

Based on these data, we hypothesized that the addition of nanogram doses of glucagon to fast-acting insulin before subcutaneous injections would speed up insulin absorption and provide a faster insulin onset of effect on glucose utilization. To test this hypothesis, we conducted a series of proof-of-concept experiments on anesthetized pigs in which glucagon was premixed with insulin lispro formulations (Lyumjev^Ⓡ^ or Humalog^Ⓡ^) and was compared with each insulin lispro formulation alone.

## Materials and Methods

### Ethics statement

The present experiments were approved by the Norwegian Food Safety Authorities (FOTS number 29687) and complied with the “Norwegian Regulation on the Use of Animals in Research” and the 2010/63 EU directive on the “Protection of Animals Used for Scientific Purposes.” For describing the experiments, we followed the ARRIVE guidelines (Animal Research: Reporting of In Vivo Experiments).^6^

### Experimental animals

We used 17 juvenile, nondiabetic, crossbred pigs (approximately 12 weeks old), obtained from the same local farmer. Fifteen were males (mean [SD] weight, 37.3 [4.8] kg) and 2 were females (mean [SD] weight, 37.0 [0.0] kg). The pigs arrived at the animal facility approximately 1 week before the experiments began. The allocation of pigs across various experimental groups and their corresponding mean weights are presented in Table 1.

**Table 1:** Overview of all experiments: control series and series with nanogram amounts of glucagon premixed in insulin solution.

Experiment number	Insulin	Group	Sex (M:F)	Treatment*	Weight (kg), mean (SD)
1	Lyumjev^Ⓡ^	Control† (n = 5)	3:2	Insulin only	39.4 (3.5)
2		Active (n = 4)	4:0	Premixed insulin	35.7 (4.6)
3	Humalog^Ⓡ^	Control (n = 4)	4:0	Insulin only	37.5 (6.5)
4		Active (n = 4)	4:0	Premixed insulin	34.5 (6.9)

F = female; M = male.

⁎ Premixed = 5.5 ng of glucagon/1 U of insulin.

† Data have been published earlier.^7^

### Housing and husbandry

Each pig was housed in an individual pen with a floor area of 2.8 m^2^, featuring a concrete floor layered with wood chips. Inter-pen connectivity was allowed when needed (maximum space, 11.2 m^2^). To ensure thermal comfort, a heating lamp was available in each pen. The facility maintained a day–night photoperiod, with nighttime spanning from 2100 to 0500, dawn from 0500 to 0700, daytime from 0700 to 1900, and dusk from 1900 to 2100. The temperature was kept constant at 22°C and the relative humidity set at 45% ± 5%.

### Animal care and monitoring

The pigs were fed a standard diet (Format Vekst 100, Felleskjøpet, Norway) and water provided ad libitum. Food was withdrawn 17 hours before the initiation of the experiment. The pigs were continuously monitored throughout the experimental procedures. All interventions, that is, surgical procedures, drug administration, and blood samplings, were performed under general anesthesia.

### Study design

In the control groups, 10 units of insulin lispro (Lyumjev^Ⓡ^ or Humalog^Ⓡ^) were injected in subcutaneous tissue. In the intervention groups, 10 units of insulin (Lyumjev^Ⓡ^ or Humalog^Ⓡ^) premixed with glucagon were administered. Data from the insulin Lyumjev^Ⓡ^ control group have been published previously in a poster comparing Lyumjev^Ⓡ^ only with Lyumjev^Ⓡ^and 0.1 mg or 0.01 mg of glucagon injections at the same subcutaneous site.^5^

### Sample size

The control group included 9 pigs; the active group included 8 pigs. Detailed information is given in Table 1.

### Inclusion and exclusion criteria

Pigs in generally good health (active, eating, and drinking) were included in the study. Pigs were excluded before the experimental phase if they showed any signs of decreased health or visible injuries (deep scratch marks and bite marks on the tail) sustained during the acclimatization period. No pigs that entered the active phase were excluded in the present set of experiments.

### Randomization

No randomization was performed in the Lyumjev or Humalog subgroups.

### Blinding

The investigators were not blinded due to the premixing of the drugs and because this was a set of proof-of-concept experiments.

### Outcome measures

The primary outcome of the study was the change in insulin plasma levels after the administration of insulin-alone boluses and insulin/glucagon boluses. An exact primary end point was not set in advance as this was a proof-of-concept design. The secondary outcome was the glucose infusion rate after the administration of insulin boluses.

### Experimental procedures

#### Premedication and anesthesia

Premedication and anesthesia were identical to those used in previous experiments.^2^^,^^8^

#### Surgical procedures

Surgical procedures were performed as in previous pig experiments.^2^^,^^9^ Intravenous fluids and drugs were administered using a venous catheter inserted in the left internal jugular vein. Blood samples were collected through an arterial catheter inserted into the left carotid artery. A small, low laparotomy was performed to expose the bladder and insert a bladder catheter for urine collection.

#### Suppression of endogenous insulin secretion

The procedure for suppression of endogenous insulin secretion was identical to previous animal experiments.^8^ The pigs received an initial intravenously administered bolus of 5 µg/kg octreotide‡ 1 hour before the administration of the insulin bolus, immediately followed by continuous intravenous infusion of 5 µg/kg/h octreotide throughout the experiment day. Previously, we verified that this procedure suppresses insulin secretion to very low or unmeasurable levels (<2.3 mU/L).^2^^,^^9^^,^^10^ However, temporarily increased glucose levels secondary to glucagon injections may increase endogenous insulin levels for a short period of time followed by a decrease even below baseline.^2^ In addition, continuous octreotide infusion suppresses endogenous glucagon secretion to unmeasurable levels (<2.18 pmol/L) in most pigs.

#### Euglycemic clamp

Before the start of each experiment, the arterial blood glucose levels were stabilized within the target range of 4.5 to 5.5 mmol/L by adjusting the intravenous infusion rate of a 20% glucose solution§. The blood glucose concentration was considered stable when the variance between last 2 samples was ≤0.2 mmol/L with no adjustments to the glucose infusion rate for 15 minutes. During the experiments, the glucose infusion rate was adjusted as needed to maintain the glucose levels within the target range of 4.5 to 5.5 mmol/L after insulin administration and throughout the subsequent 210 minutes of the experiment.

#### Preparing the solution of insulin premixed with glucagon

Insulin was premixed with glucagon in 10-mL commercial insulin vials (Lyumjev or Humalog with insulin concentrations of 100 U/mL). We used 1 mg glucagon powder (GlucaGen HypoKit, NovoNordisk AS, Bagsværd, Denmark) dissolved in the 1 mL solution provided by the manufacturer to achieve a concentration of 1 mg/mL glucagon. Later, 0.5 mL of this glucagon solution was mixed with 99.5 mL 0.9% saline, reaching a glucagon concentration of 0.005 mg/mL. Then, 1 mL of this glucagon solution was added to a 10-mL insulin vial, yielding a mixture with 5 ng glucagon/1 unit of insulin. The addition of 1 mL solution to the 10-mL insulin vial reduced the insulin concentration to 90.91 U/mL. Therefore, to deliver a dose of 10 units of insulin, 0.11 mL of premixed solution was injected. The solutions were stored in a refrigerator for 3 to 7 days before being used. The stability of this solution was verified (unpublished data).

#### Insulin/glucagon bolus

In the control group, immediately after blood sampling at time 0, each pig received a 60-second subcutaneous infusion of 10 units insulin lispro (Lyumjev^Ⓡ^ or Humalog^Ⓡ^). In the glucagon group, the same insulin (10 U) premixed with glucagon (50 ng) was infused. Infusions were performed behind the left ear using a Chemyx Fusion 100 syringe pump and a 9-mm needle (Precision Glide needle 30 gauge × 1 inch (0.3 mm × 25 mm); Becton Dickinson, Franklin Lakes, New Jersey), inserted in subcutaneous tissue at a 45° angle using a custom-made insertion guide to ensure the same depth of subcutaneous injection in all pigs.

#### Blood sampling

Thirty minutes after the start of octreotide infusion, a blood sample was collected to verify endogenous insulin suppression. Thereafter, blood samples were collected at time points 0, 1, 5, 10, 15, 20, 25, 30, 35, 40, 50, 60, 90, 120, 150, 180, and 210 minutes. Additional blood samples were drawn as needed to adjust the euglycemic clamp glucose infusion rate. To include at least 2 predicted half-lives of insulin lispro, we set the length of the total experiment to 210 minutes.

#### Sample handling and analysis

Arterial blood glucose concentrations were analyzed immediately after collection using a Radiometer ABL 800 FLEX blood gas analyzer (Radiometer Medical ApS, Brønshøj, Denmark) to guide the euglycemic clamp procedure. Both the intra-assay and the interassay coefficients of variation for glucose analyses were <5%.

Arterial blood samples for insulin analyses were collected into 3-mL EDTA vacutainers and kept on ice for 10 minutes before centrifugation. Afterwards, the plasma was transferred to 2 Eppendorf tubes (1.5 mL, Invitrogen, Life Technologies, CA, USA) and stored at −18°C for the rest of the experiment day and transferred to a −80°C freezer at the end of the day until analysis. These analyses were performed within 7 days.

Insulin levels were analyzed by the same laboratorian (I.D.-F.) using the Iso-Insulin ELISA kit 10-1128-01 (Mercodia, Uppsala, Sweden); the lower detection limit was 2.0 mU/L. In the results, all values <2.0 mU/L were set to 1.0 mU/L. All samples were run in doubles. The ELISA kits for iso-insulin were all from the same batch. The intra-assay and interassay variations were both <8%.

### Pharmacokinetic analysis

Free serum insulin lispro pharmacokinetic parameters were calculated by linear trapezoidal methods using Microsoft 365 Excel version 2311 and GraphPad Prism version 10.2.0. We determined the primary end points to be early exposure AUC from time 0 to15 minutes (AUC_0–15_), to 30 minutes (AUC_0–30_), and to 1 hour (AUC_0–60_) and overall exposure to AUC from time 0 to last measurement (AUC_0–210_), C_max_, and T_max_. C_max_ and T_max_ were obtained using the time and concentration curve, that is, the highest insulin level in each pig and the time when the highest insulin concentration was measured.

Secondary end points were all other AUC measured at included time points during the study.

### Statistical methods

Adjustment for baseline (time 0) insulin levels were performed for all insulin levels measured during the experiments. Means and SE of differences for T_max_, C_max_, and AUC with 95% CI were used to compare groups, if not mentioned otherwise. Time point 0 was used as baseline for each pig. The means and range of free insulin concentration, glucose infusion rate, and AUC of insulin were calculated using GraphPad Prism 10. The statistical differences in insulin concentrations, T_max_, C_max_, and AUC between the groups were analyzed by using 2-sample *t* test for unpaired variance. No adjustment for multiple testing was performed, and a *P* value <0.05 was considered significant.

## Results

### Suppression of endogenous insulin

Before any intervention, the free insulin level was below the detection limit of 2.00 mU/L in 5 of 17 pigs. In the remaining 12 pigs, the mean (SD) free insulin level was 6.47 (4.30) mU/L, ranging from 2.43 to 18.96 mU/L. At time point 0, free insulin levels were below the detection limit of 2.00 mU/L in 5 of 17 pigs. In the remaining 12 pigs, the mean (SD) free insulin level was 5.73 (4.60) mU/L, ranging from 2.23 to 19.12 mU/L. The baseline insulin value in each pig was considered as the endogenous insulin level during the study and thus subtracted from later measured insulin levels. Thus, the delta insulin values are the change from baseline.

### Pharmacokinetics

#### Insulin Lyumjev and Humalog concentration–time profiles

**Fig. 1:**
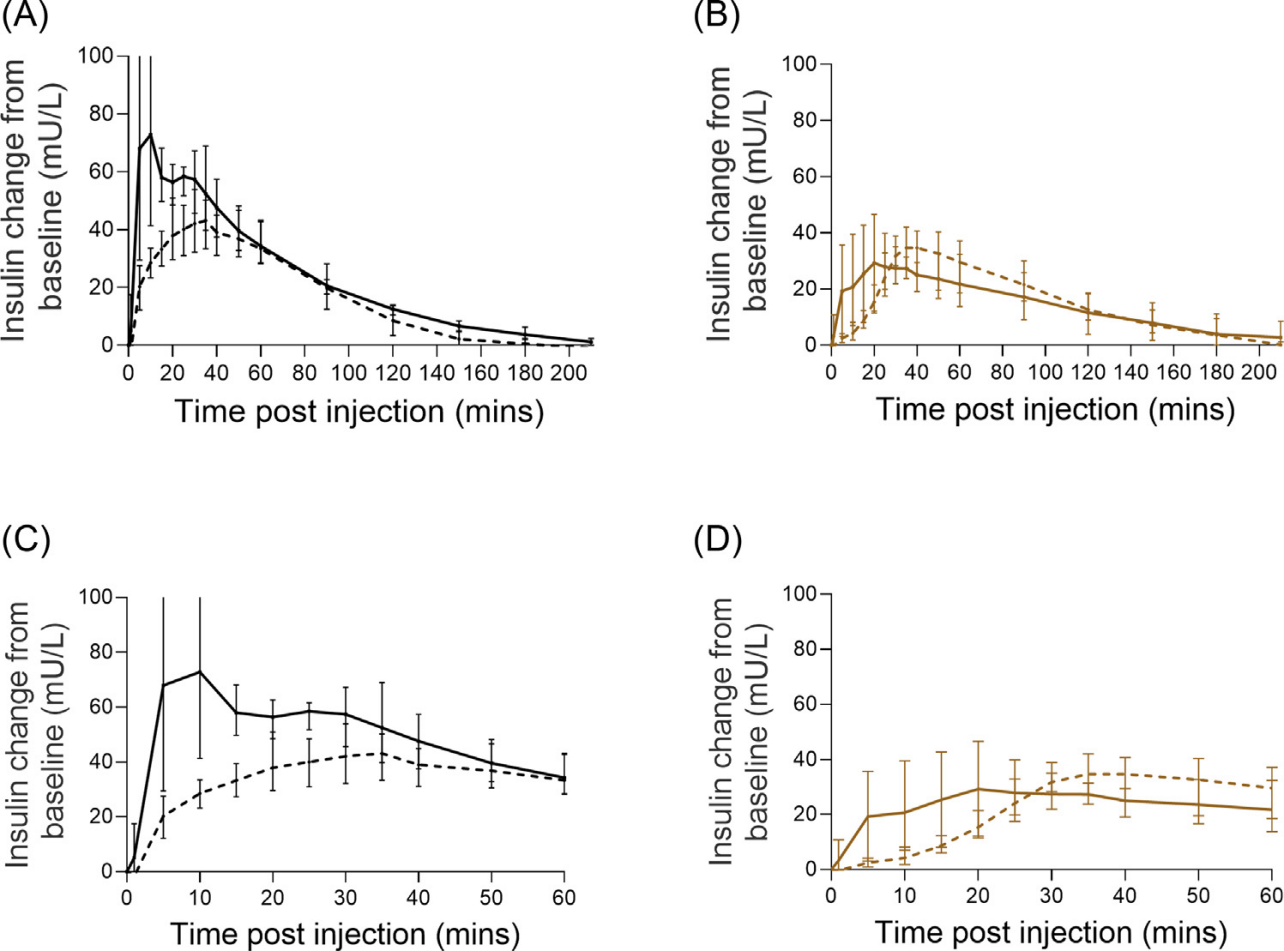
Mean (± range) serum insulin lispro concentration–time change from baseline for Lyumjev^Ⓡ^ (black dashed line) and Humalog^Ⓡ^ (brown dashed line) and premixed Lyumjev^Ⓡ^/glucagon (black line) and Humalog^Ⓡ^/glucagon (brown line). (A) 0 to 210 minutes after injection of Lyumjev^Ⓡ^; (B) 0 to 210 minutes after injection of Humalog^Ⓡ^; (C) 0 to 60 minutes after injection of Lyumjev^Ⓡ^; (D) 0 to 60 minutes after injection of Humalog^Ⓡ^.

Mean insulin Lyumjev^Ⓡ^ and Humalog^Ⓡ^ concentrations appeared visually higher at earlier time points after the premixed Lyumjev^Ⓡ^/glucagon or Humalog^Ⓡ^/glucagon injections, compared with their respective control groups (Figure 1A and 1b), indicating a faster insulin absorption and earlier decline in insulin concentrations. For premixed Lyumjev^Ⓡ^, insulin concentrations not only increased earlier but also reached a higher peak (Table 2).

**Table 2:** Pharmacokinetic variables of insulin Lyumjev^Ⓡ^.

Variable	Lyumjev^Ⓡ^ control* (n = 5)†	Lyumjev^Ⓡ^/glucagon‡ (n = 4)	*P* value
T_max_ (min)	33 (30–35)	20 (5–35)	0.090
C_max_ (mU/L)	45 (32–59)	100 (51–150)	**0.030**
AUC_0–15_ (mU×min/L)	337 (226–449)	946 (252–1640)	**0.017**
AUC_0–30_ (mU×min/L)	932 (629–1234)	1922 (1321–2523)	**0.002**
AUC_0–60_ (mU×min/L)	2106 (1551–2661)	3433 (3171–3695)	**0.0008**

T_max_ and C_max_ were obtained directly from real time data. AUC was calculated as net area. Values in parentheses for T_max_ are minimum to maximum values; C_max_ values are delta mean (95% CI); values in parentheses for AUC are 95% CI.

⁎ Each pig received a 60-second subcutaneous infusion of 10 U of insulin.

† Data have been published earlier.^7^

‡ Insulin (10 U) was premixed with glucagon (55 ng).

##### Early insulin Lyumjev exposure

Insulin Lyumjev^Ⓡ^ concentration was significantly higher at 10 to 30 minutes in the premixed insulin Lyumjev^Ⓡ^/glucagon group compared with the control group (Figure 1C and 1D; Supplemental Table 1).

As shown in Figure 1 and Table 2, the insulin T_max_ was observed to be earlier (20 minutes [95% CI, −3.4 to 43.4] vs 33 minutes [95% CI, 29.6–36.4]; *P* = 0.09), while C_max_ was 117% increased (100 mU/L [95% CI, 50.8–149.8] vs 46 mU/L [95% CI, 32.1–59.4]; *P* = 0.007) in the premixed Lyumjev^Ⓡ^/glucagon group compared with the control group. Higher C_max_ was observed in all 4 pigs in the premixed Lyumjev^Ⓡ^/glucagon group (Supplemental Figure 1).

This faster insulin Lyumjev^Ⓡ^/glucagon absorption led to significantly increased insulin Lyumjev^Ⓡ^ concentration in the first 15 minutes as insulin AUC_0–15_ was 180% higher (946 mU×min/L [95% CI, 252–1640] vs 337 mU×min/L [95% CI, 226–449]; *P* = 0.02) in the insulin Lyumjev^Ⓡ^/glucagon group compared with the control group (Table 2; Supplemental Table 2).

##### Early insulin Humalog exposure

For Humalog^Ⓡ^, there was no difference between premixed Humalog^Ⓡ^/glucagon injection and the control group for any of the variables studied (Table 3; Supplemental Tables 3 and 4; Figure 1).

**Table 3:** Pharmacokinetic variables of insulin Humalog^Ⓡ^.

Variable	Humalog^Ⓡ^ control* (n = 4)†	Humalog^Ⓡ^/glucagon‡ (n = 4)	*P* value
T_max_ (min)	36 (30 to 40)	31 (20 to 50)	0.613
C_max_ (mU/L)	37 (20 to 59)	38 (20 to 56)	0.839
AUC_0–15_ (mU×min/L)	64 (−4.8 to 134)	306(−86 to 697)	0.102
AUC_0–30_ (mU×min/L)	398 (113 to 684])	770 (35 to 1503)	0.184
AUC_0–60_ (mU×min/L)	1493 (624 to 2363)	1563 (719 to 2408)	0.860

T_max_ and C_max_ were obtained directly from real time data. AUC was calculated as net area. Values in parentheses for T_max_ are minimum to maximum values; C_max_ values are delta mean (95% CI); values in parentheses for AUC are 95% CI.

⁎ Each pig received a 60-second subcutaneous infusion of 10 U of insulin.

† Data have been published earlier.^7^

‡ Insulin (10 U) was premixed with glucagon (55 ng).

#### Euglycemic clamp investigations

Blood glucose levels were measured every 5 to 10 minutes during periods with blood glucose changes due to the injected insulin and at least every 15 minutes during other periods. To keep the blood glucose levels in the desired range (4.5–5.5 mmol/L), the intravenous glucose infusion rate was adjusted as needed (euglycemic clamp) (Figure 3; Supplemental Figure 4).

**Fig. 2:**
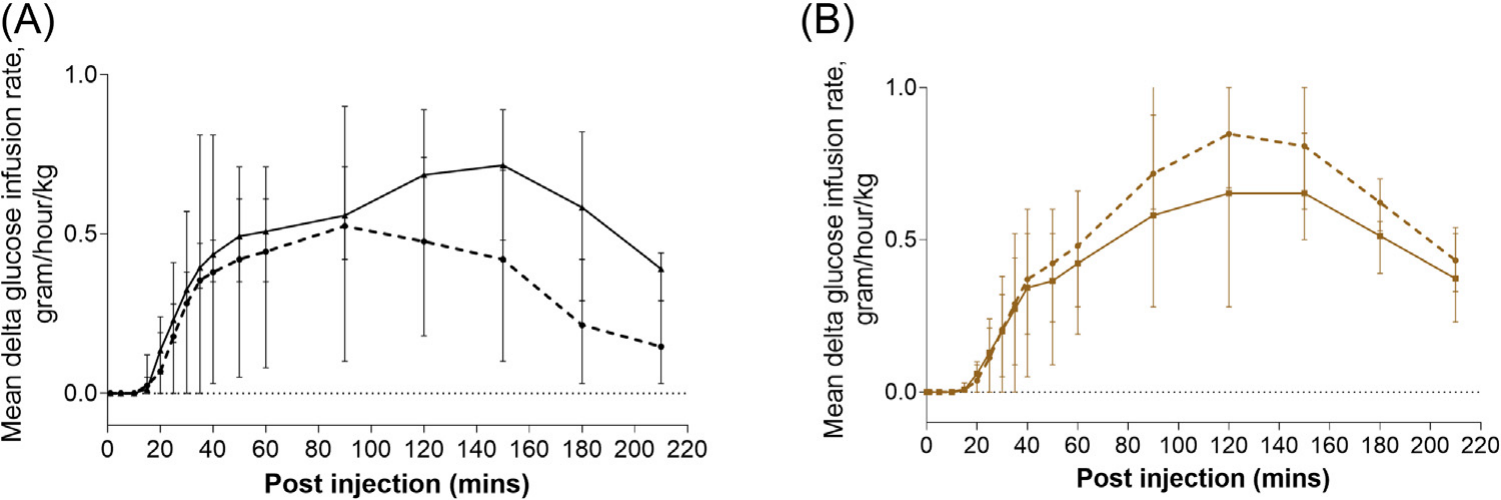
Glucose infusion rate change from baseline (± range) after insulin (dashed line) and premixed insulin/glucagon injection (solid line); (A) 0 to 210 minutes after injection of Lyumjev^Ⓡ^; (B) 0 to 210 minutes after injection of Humalog^Ⓡ^.

We observed similar glucose infusion rates, that is, T_max_ and C_max_, in the insulin Lyumjev^Ⓡ^ and the insulin Humalog^Ⓡ^ groups (Figure 2; Tables 4 and 5, and Supplemental Tables 5-7). There was similar glucose utilization in all the compared groups with AUC not statistically different at any of the time points (Tables 4 and 5; Supplemental Tables 8 and 9; Supplemental Figure 5).

**Table 4:** Variables of glucose infusion rate after insulin Lyumjev^Ⓡ^.

Variable	Lyumjev^Ⓡ^ control* (n = 5)†	Lyumjev^Ⓡ^/glucagon‡ (n = 4)	*P* value
T_max_ (min)	102 (60 to 120)	115 (40 to 150)	0.640
C_max_ (mU/L)	0.6 (0.2 to 0.9)	0.7 (0.4 to 1.0)	0.387
AUC_0–15_ (mU×min/L)	0.1 (−0.1 to 0.2)	0.03 (−0.1 to 0.1)	0.707
AUC_0–30_ (mU×min/L)	2.1 (−0.5 to 4.6)	2.7 (1.7 to 3.7)	0.566
AUC_0–60_ (mU×min/L)	13.8 (2.1 to 25.5)	16.2 (11.9 to 20.5)	0.639

T_max_ and C_max_ were obtained directly from real time data. AUC was calculated as net area. Values in parentheses for T_max_ are minimum to maximum values; C_max_ values are delta mean (95% CI); values in parentheses for AUC are 95% CI.

⁎ Each pig received a 60-second subcutaneous infusion of 10 U of insulin.

† Data have been published earlier.^7^

‡ Insulin (10 U) was premixed with glucagon (55 ng).

**Table 5:** Variables of glucose infusion rate after insulin Humalog^Ⓡ^.

Variable	Humalog^Ⓡ^ control* (n = 4)†	Humalog^Ⓡ^/glucagon‡ (n = 4)	*P* value
T_max_ (min)	113 (90 to 120)	120 (90 to 150)	0.620
C_max_ (mU/L)	0.9 (0.6 to 1.1)	0.7 (0.4 to 1.0)	0.292
AUC_0–15_ (mU×min/L)	0.02 (−0.04 to 0.1)	0.02 (−0.04 to 0.08)	>0.999
AUC_0–30_ (mU×min/L)	1.3 (−0.5 to 3.1)	1.49 (−0.3 to 3.3)	0.821
AUC_0–60_ (mU×min/L)	12.7 (4.2 to 21.1)	11.7 (−0.1 to 23.5)	0.837

T_max_ and C_max_ were obtained directly from real time data. AUC was calculated as net area. Values in parentheses for T_max_ are minimum to maximum values; C_max_ values are delta mean (95% CI); values in parentheses for AUC are 95% CI.

⁎ Each pig received a 60-second subcutaneous infusion of 10 U of insulin.

† Data have been published earlier.^7^

‡ Insulin (10 U) was premixed with glucagon (55 ng).

## Discussion

Our hypothesis that premixing nanogram doses of glucagon with commercially available insulin lispro formulations (Lyumjev^Ⓡ^ and Humalog^Ⓡ^) enhances the first phase of insulin absorption is partially supported by the observations in the present set of proof-of-concept experiments. Figure 2A shows a marked increase in plasma insulin Lyumjev^Ⓡ^ levels during the first 15 minutes after the injection of premixed insulin Lyumjev^Ⓡ^/glucagon solution compared with the Lyumjev^Ⓡ^-only injection. Notably, within 10 minutes of injecting the premixed insulin Lyumjev^Ⓡ^/glucagon solution, the insulin level was comparable to the maximum insulin concentration reached 40 minutes after the Lyumjev-only injections. The main effect of fast-acting mealtime insulin is to rapidly saturate the liver with insulin to inhibit hepatic glucose release and promote hepatic glucose uptake. We believe that an approximately 3-fold increase of insulin AUC_0–15_ after the premixed insulin Lyumjev^Ⓡ^ injection corresponds to an equally faster hepatic insulin saturation.

Local subcutaneous blood flow and subcutaneous absorption of insulin are typically correlated, and we hypothesize that adding glucagon to the insulin formulations enhances the overall insulin absorption by promoting local vasodilation.^11^^,^^12^ In the present pig experiments, the insulin absorption varied substantially regardless of whether insulin was administered alone or as a premixed insulin–glucagon solution. Similarly, significant interindividual variation in response to the glucagon injections was also observed in our previous studies.^2^^,^^8^^,^^13^ The variability in insulin absorption observed in this series of pig experiments resembles the day-to-day variation in insulin absorption and glucose response seen in humans, even those with consistent lifestyles and insulin regimens.

To add glucagon to insulin solutions to speed up the absorption of subcutaneously injected insulin is innovative and counterintuitive. Many would argue against the use of glucagon to enhance insulin absorption, as glucagon exerts antagonistic effects on glucose metabolism and increases blood glucose levels. However, we believe that the glucagon doses used in these experiments are far too small to exert any adverse effect on glucose levels in humans. Endogenous glucagon is present in the blood of both healthy subjects and patients with T1D. Moreover, data also indicates that glucagon levels increase during meals, probably due to release from the intestine.^14^ In this scenario, the additional nanogram doses of exogenous glucagon, delivered premixed with insulin, are likely neglectable.

However, we did not observe a faster or overall increased effect on glucose utilization (the euglycemic clamp investigations). It is important to note that, during these experiments, endogenous glucagon secretion was suppressed by an injection, followed by a continuous infusion of a somatostatin analogue. This opens the possibility that, in the present experimental setting, even nanogram doses of glucagon may exert an adverse effect on hepatic glucose metabolism when endogenous glucagon is absent/suppressed. As mentioned, this would hardly be the case in awake patients with T1D, particularly after a meal, when endogenous glucagon levels increase significantly (Dirnena-Fusini I, Riaz M, Christiansen SC, Carlsen SM. Importance of exact same site glucagon and insulin injection to enhance insulin absorption: Results of human proof-of-concept RCT. Submitted for publication).

We focused our proof-of-concept experiments on adding nanogram doses of glucagon to Lyumjev^Ⓡ^, the most fast-acting insulin formulation available on the market. As previously discussed, we believe glucagon may increase insulin absorption through significant but very localized subcutaneous vasodilation. Therefore, it is noteworthy that when glucagon was added to Lyumjev^Ⓡ^, an insulin formulation that already contains 10 ng of the vasodilator treprostinil per unit of insulin, we observed even faster insulin absorption. This effect was not observed in experiments with glucagon added to Humalog^Ⓡ^. This may indicate that there is an interaction between glucagon and treprostinil in terms of vasodilative effect. We know that glucagon enhances subcutaneous blood flow by 4% to 500 % in healthy subjects.^4^ If the present effect of adding nanogram amounts of glucagon to insulin Lyumjev also is present in humans and translates to a faster effect on glucose levels, this could be the next improvement in the treatment of T1D. Faster insulin absorption may transform a hybrid artificial pancreas (AP) into a fully automated AP, fulfilling the long-awaited solution, a fully automated AP with good glucose control and no need for daily input from patients.

### Future studies

The next logical step will be to study the effect of premixing nanograms of glucagon in insulin Lyumjev^Ⓡ^ and insulin Humalog^Ⓡ^ in patients with T1D.

### Limitations

A major limitation of these experiments is that anesthesia in pigs might influence the metabolism in the pigs.^15^ Hence, there is a possibility that anesthesia influences the pharmacokinetic and pharmacodynamic properties of insulin. Duration of experiments may also have influenced the response. In previous pig studies, we observed accumulation of fluids in the body after prolonged anesthesia. Experiments on conscious pigs would have increased the reliability of the data. However, after ethical considerations, drawing blood samples every 5 minutes from conscious pigs was deemed not acceptable.

Another limitation is the use of a somatostatin analogue to suppress endogenous insulin secretion and induce insulin deficiency, that is, a model of T1D. We followed a protocol that provided adequate suppression in a previous pig study.^13^ Nevertheless, in many pigs a full suppression of insulin was not achieved, while glucagon was suppressed to unmeasurable levels in most pigs. Measured insulin levels were adjusted for baseline insulin level in each individual pig, but, as we observed in previous studies,^2^ endogenous insulin levels decrease during the somatostatin analog infusion. Thus, reported insulin levels may be biased, as we have no data of change of endogenous insulin level.

Maintaining glucose range (4.5–5.5 mmol/L) was challenging (Supplemental Figure 6). Thus, glucose infusion rate may be a biased evaluation of glucose utilization in the pigs.

Finally, these experiments had a very small sample size, which opens the possibility for both type 1 and type 2 statistical errors. Given the wide variation in the speed of insulin absorption both in native insulins and insulins premixed with glucagon, this is a major limitation. The strength of these experiments is frequent blood samples for glucose and insulin measurements.

## Conclusions

This series of proof-of-concept experiments on anesthetized pigs indicate that premixing nanogram doses of glucagon with insulin Lyumjev^Ⓡ^ may speed up the subcutaneous absorption of insulin. Regardless of treatment modality, increasing insulin absorption by adding nanogram doses of glucagon to insulin Lyumjev^Ⓡ^ formulation could improve glucose control in patients with T1D. It may even improve glucose control in hybrid AP by increasing time in range (glucose 3.9–10.0 mmol/L). Most notable, it may also facilitate the development of a fully automated AP with good glucose control.

## Funding

The experiment was funded by the Research Council of Norway (grant number 332850) and the Central Norway Regional Health Authority (grant number 2020/39645).

## Declaration of competing interest

The authors declare the following financial interests/personal relationships which may be considered as potential competing interests:

The authors declare that the research at the time was conducted without any commercial or financial relationships that could be construed as a potential conflict of interest. NTNU, the university where the research was conducted and where the researchers reside, has a patent filed related to the research. The authors Sven Magnus Carlsen and Sverre Christian Christiansen are among the inventors. The remaining authors have no conflicts of interest to declare.

## Appendix Supplementary materials

Image, application 1

## Footnotes

⁎ Trademark: Lyumjev^Ⓡ^ (Eli Lilly, Norway AS, Norway).

† Trademark: Humalog^Ⓡ^ (Eli Lilly, Norway AS, Norway).

‡ Trademark: Sandostatin (Novartis Europharm Limited, London, United Kingdom).

§ Trademark: Glucos B. Braun (B. Braun Melsungen AG, Melsungen, Germany).

## References

[1] Robinson S., Newson R.S., Liao B. (2021). Missed and mistimed insulin doses in people with diabetes: a systematic literature review. Diabetes Technol Ther.

[2] Teigen I.A., Åm M.K., Riaz M. (2024). Effects of low-dose glucagon on subcutaneous insulin absorption in pigs. Curr Ther Res Clin Exp.

[3] Heise T., Linnebjerg H., Coutant D. (2020). Ultra rapid lispro lowers postprandial glucose and more closely matches normal physiological glucose response compared to other rapid insulin analogues: a phase 1 randomized, crossover study. Diabetes Obes Metab.

[4] Åm M.K., Munkerud E.Y., Berge M.H. (2022). The effect of glucagon on local subcutaneous blood flow in non-diabetic volunteers; a proof-of-concept study. Eur J Pharmacol.

[5] Carlsen S.M., Christiansen SC. (2023). 16th International Conference on Advanced Technologies & Treatments for Diabetes; February 22–25.

[6] Percie du Sert N., Hurst V., Ahluwalia A. (2020). The ARRIVE guidelines 2.0: updated guidelines for reporting animal research. PLoS Biol.

[7] Christiansen S.C., Fougner A.L., Stavdahl Ø (2017). A review of the current challenges associated with the development of an artificial pancreas by a double subcutaneous approach. Diabetes Ther.

[8] Teigen I.A., Åm M.K., Carlsen S.M., Christiansen SC. (2021). Pharmacokinetics of intraperitoneally delivered glucagon in pigs: a hypothesis of first pass metabolism. Eur J Drug Metab Pharmacokinet.

[9] Åm M.K., Dirnena-Fusini I., Fougner A.L. (2020). Intraperitoneal and subcutaneous glucagon delivery in anaesthetized pigs: effects on circulating glucagon and glucose levels. Sci Rep.

[10] Dirnena-Fusini I., Åm M.K., Fougner A.L. (2021). Intraperitoneal insulin administration in pigs: effect on circulating insulin and glucose levels. BMJ Open Diabetes Res Care.

[11] Binder C. (1969). Absorption of injected insulin. A clinical-pharmacological study. Acta Pharmacol Toxicol (Copenh).

[12] Vora J.P., Burch A., Peters J.R., Owens DR. (1992). Relationship between absorption of radiolabeled soluble insulin, subcutaneous blood flow, and anthropometry. Diabetes Care.

[13] Teigen I.A., Åm M.K., Carlsen S.M., Christiansen SC. (2022). Pharmacokinetics of glucagon after intravenous, intraperitoneal and subcutaneous administration in a pig model. Basic Clin Pharmacol Toxicol.

[14] Ito A., Horie I., Miwa M. (2021). Impact of glucagon response on early postprandial glucose excursions irrespective of residual β-cell function in type 1 diabetes: a cross-sectional study using a mixed meal tolerance test. J Diabetes Investig.

[15] Slupe A.M., Kirsch JR. (2018). Effects of anesthesia on cerebral blood flow, metabolism, and neuroprotection. J Cereb Blood Flow Metab.

