# Supplementary material for: Nano-Amounts of Glucagon Premixed With Fast-Acting Insulin Lispro: Effect on Insulin Absorption in Pigs
Source: Curr Ther Res Clin Exp. 2025 Jun 24;103:100803. doi: 10.1016/j.curtheres.2025.100803 (PMC12296474; doi:10.1016/j.curtheres.2025.100803)
Supplement: Supplementary file 1 [file mmc1.docx]

Supplementary materials for article **“Nano-amounts of glucagon pre-mixed with fast-acting insulin lispro: Effect on insulin absorption in pigs”**

**INSULIN ANALYSES**

**Insulin Lyumjev^®^ and insulin Humalog^®^**


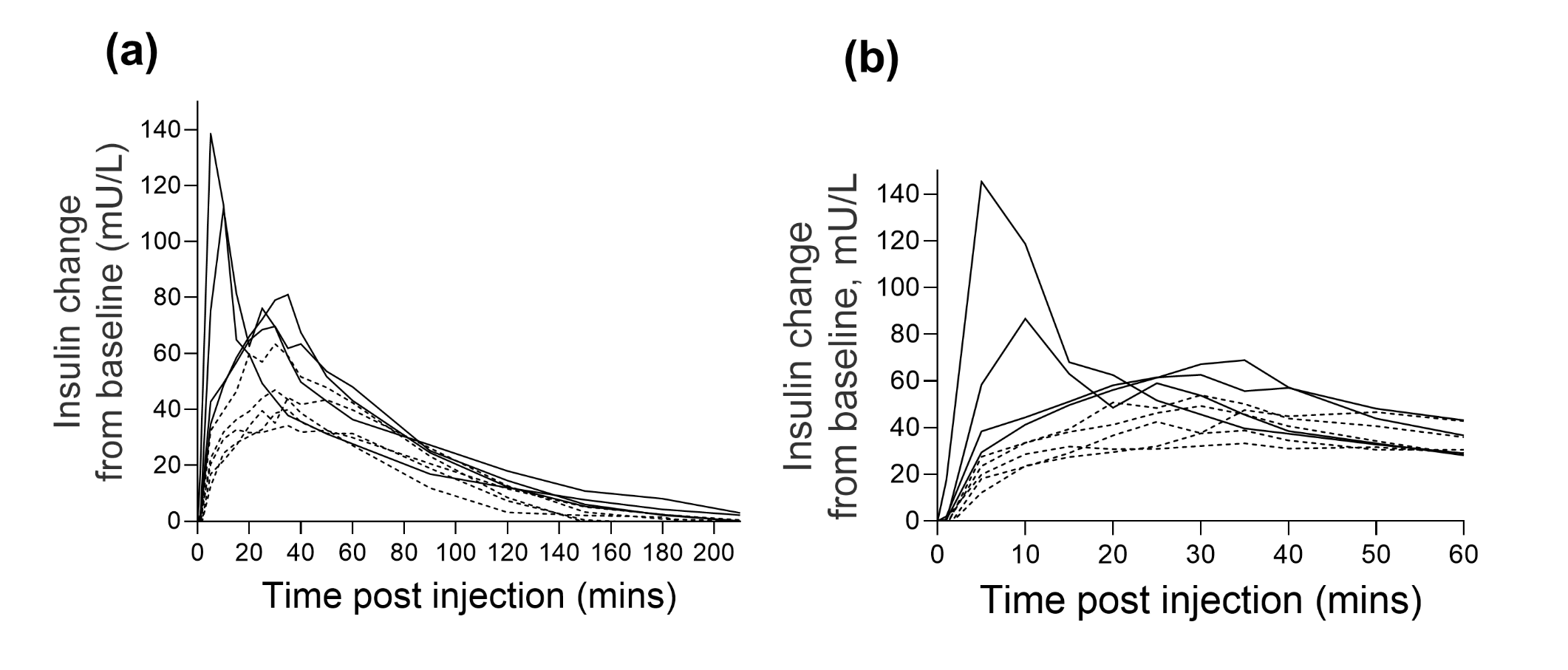


**Supplemental Figure 1.** Individual data for insulin change from baseline after insulin Lyumjev (black dashed lines) and premixed insulin Lyumjev/glucagon (black solid lines). (**a**) all period of study time post injection, (**b**) first 60 minutes post injection.


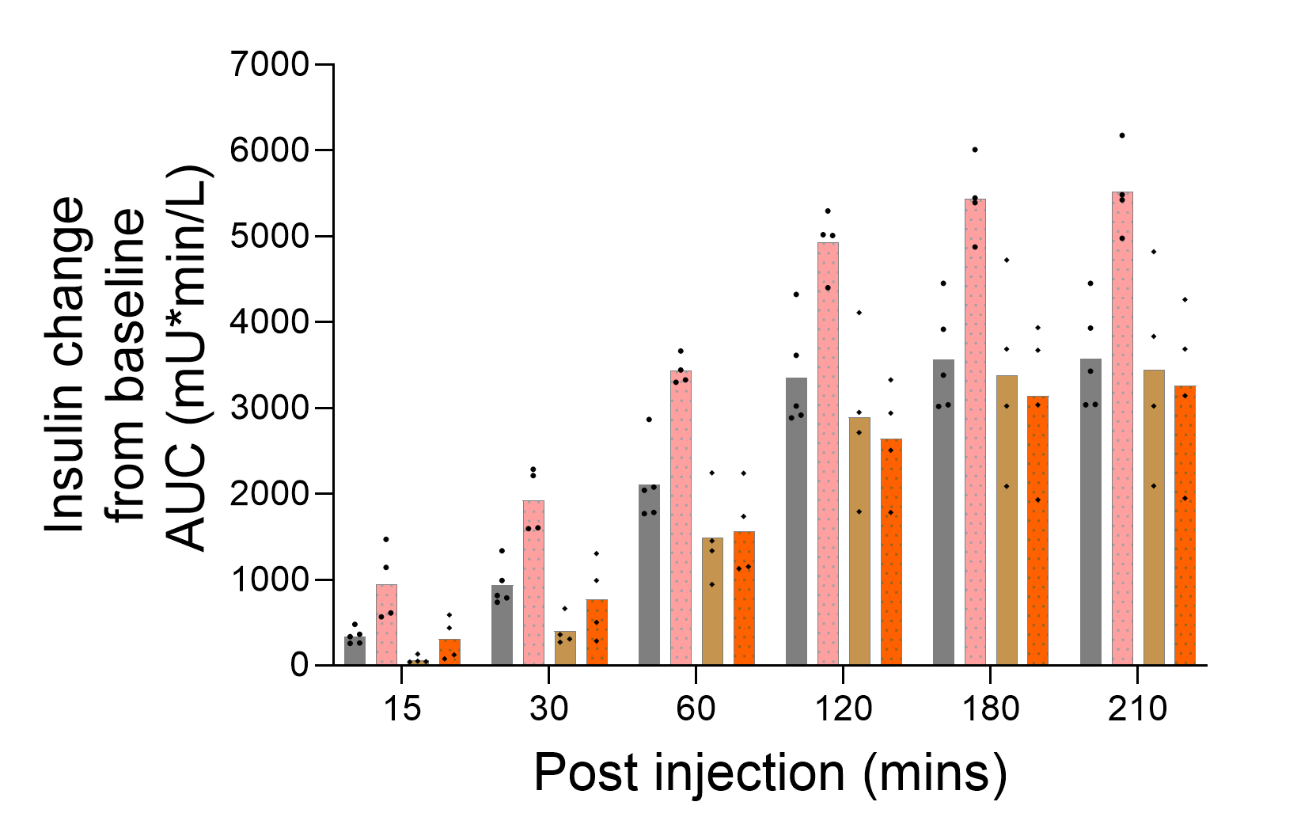


**Supplemental Figure 2**. Insulin mean change from baseline, Area Under the Curve (AUC) summary for all series of experiments. Grey column, Control group (insulin Lyumjev); pink with dotes column, Glucagon group (pre-mixed insulin Lyumjev/Glucagon); Brown column, control group (insulin Humalog); orange with dotes column, glucagon group (pre-mixed insulin Humalog/Glucagon). Each dote on the columns represent individual pig.


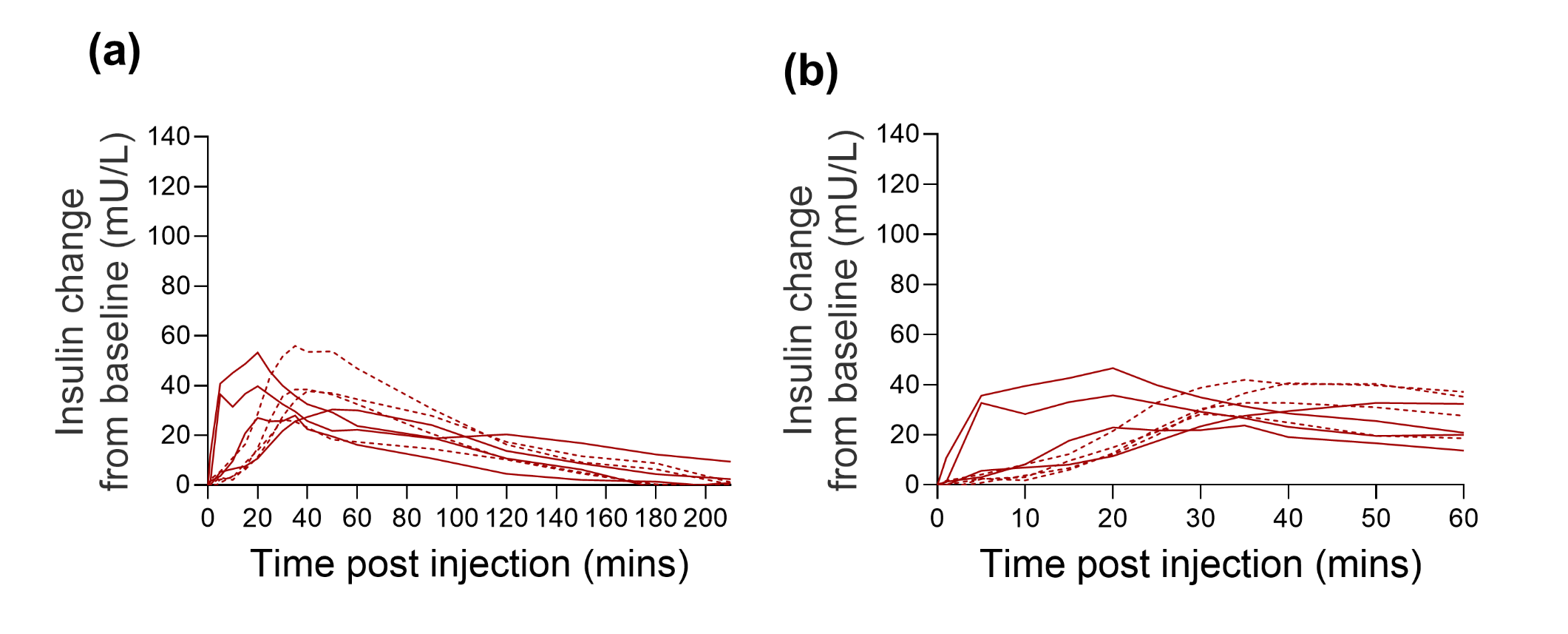


**Supplemental Figure 3.** Individual data for insulin change from baseline after insulin Humalog (red dashed lines) and premixed insulin Humalog/glucagon (red solid lines). (**a**) all period of study time post injection, (**b**) first 60 minutes post injection.

**Supplemental Table 1.** Mean delta insulin Lyumjev (mU/L) difference*

| Post injection (minutes) | Mean  (glucagon group) | 95% CI | Mean  (control group) | 95% CI | Difference | T ratio | P-value |
| --- | --- | --- | --- | --- | --- | --- | --- |
| 1 | 4.95 | -7.58 - 17.48 | -1.82 | -4.15 - 0.45 | 6.80 | 1.897 | 0.099 |
| 5 | 72.83 | -2.26 - 147.9 | 20.93 | 11.68 - 30.18 | 51.90 | 2.464 | **0.043** |
| 10 | 80.74 | 22.42 - 139.1 | 29.36 | 20.73 - 37.99 | 51.38 | 3.118 | **0.017** |
| 15 | 65.39 | 47.53 - 83.26 | 34.15 | 24.48 - 43.82 | 31.24 | 4.945 | **0.001** |
| 20 | 63.25 | 58.77 - 67.73 | 39.07 | 24.03 - 54.11 | 24.18 | 3.860 | **0.006** |
| 25 | 66.52 | 47.55 - 85.50 | 41.04 | 28.35 - 53.73 | 25.49 | 3.459 | **0.011** |
| 30 | 65.42 | 41.08 - 89.77 | 43.39 | 28.02 -58.76 | 22.03 | 2.396 | **0.047** |
| 35 | 59.86 | 31.72 - 88.01 | 44.17 | 32.74 - 55.61 | 15.69 | 1.731 | 0.130 |
| 40 | 54.10 | 31.15 - 77.05 | 39.90 | 30.53 - 49.26 | 14.21 | 1.920 | 0.096 |
| 50 | 44.86 | 28.63 - 61.09 | 37.54 | 28.16 - 46.93 | 7.32 | 1.242 | 0.254 |
| 60 | 38.80 | 24.74 - 52.86 | 34.09 | 25.89 - 42.29 | 4.71 | 0.919 | 0.388 |
| 90 | 23.48 | 16.26 - 30.70 | 20.18 | 13.50 - 26.86 | 3.30 | 0.977 | 0.361 |
| 120 | 14.15 | 9.76 - 18.54 | 8.63 | 3.93 - 13.33 | 5.52 | 2.435 | **0.045** |
| 150 | 7.49 | 3.57 - 11.40 | 2.10 | -0.64 - 4.84 | 5.39 | 3.464 | **0.011** |
| 180 | 4.26 | -0.09 - 8.61 | 0.42 | -1.75 - 2.59 | 3.83 | 2.569 | **0.037** |
| 210 | 1.31 | -1.14 - 3.75 | -0.96 | -3.54 - 1.62 | 2.27 | 1.812 | 0.113 |

*Multiple Unpaired T test. 95% confidence interval (95% CI).

**Supplemental Table 2.** Mean delta insulin Lyumjev net AUC^#^ (mU^×^min/L) difference*

| Post injection (minutes) | Mean (glucagon group) | 95% CI | Mean (control group) | 95% CI | Difference | T ratio | P-value |
| --- | --- | --- | --- | --- | --- | --- | --- |
| 1 | 2.47 | -3.79 - 8.74 | 0.12 | -0.21 - 0.44 | 2.36 | 1.359 | 0.216 |
| 5 | 196.9 | -25.00 - 418.9 | 53.03 | 29.91 - 76.14 | 143.9 | 2.322 | 0.053 |
| 10 | 580.9 | 45.48 - 1116 | 178.8 | 112.0 - 245.5 | 402.1 | 2.676 | **0.031** |
| 15 | 946.2 | 252.32 - 1640 | 337.5 | 225.7 - 449.4 | 608.7 | 3.092 | **0.018** |
| 20 | 1268 | 558.4 - 1977 | 520.6 | 349.5 - 691.7 | 747.2 | 3.594 | **0.008** |
| 25 | 1592 | 920.8 - 2264 | 720.8 | 484.5 - 957.1 | 871.4 | 4.171 | **0.004** |
| 30 | 1922 | 1321 - 2523 | 931.9 | 629.2 - 1235 | 990.2 | 4.787 | **0.002** |
| 35 | 2235 | 1727 - 2743 | 1151 | 785.3 - 1516 | 1085 | 5.296 | **0.001** |
| 40 | 2520 | 2102 - 2938 | 1361 | 948.7 - 1773 | 1159 | 5.680 | **0.0007** |
| 50 | 3015 | 2719 - 3311 | 1748 | 1258 - 2238 | 1267 | 5.863 | **0.0006** |
| 60 | 3433 | 3171 - 3695 | 2106 | 1551 - 2661 | 1327 | 5.577 | **0.0008** |
| 90 | 4368 | 3925 - 4810 | 2920 | 2228 - 3613 | 1447 | 4.699 | **0.002** |
| 120 | 4932 | 4332 - 5532 | 3352 | 2584 - 4121 | 1580 | 4.453 | **0.003** |
| 150 | 5257 | 4582 - 5931 | 3515 | 2732 - 4297 | 1742 | 4.709 | **0.002** |
| 180 | 5433 | 4695 - 6171 | 3562 | 2797 - 4327 | 1871 | 5.017 | **0.002** |
| 210 | 5516 | 4727 - 6306 | 3578 | 2820 - 4335 | 1939 | 5.122 | **0.001** |

*Multiple Unpaired T test. 95% confidence interval [95% CI].

^#^Area Under the Curve (AUC).

**Supplemental Table 3.** Mean delta insulin Humalog (mU/L) difference*

| Post injection (minutes) | Mean (glucagon group) | 95% CI | Mean  (control group) | 95% CI | Difference | T ratio | P-value |
| --- | --- | --- | --- | --- | --- | --- | --- |
| 1 | 3.75 | -5.47 - 12.97 | -0.45 | -3.22 - 2.73 | 3.99 | 1.312 | 0.238 |
| 5 | 21.49 | -10.03 - 53.01 | 2.88 | -0.21 - 5.97 | 18.61 | 1.870 | 0.111 |
| 10 | 23.15 | -6.05 - 52.35 | 4.86 | -1.62 - 11.33 | 18.30 | 1.947 | 0.100 |
| 15 | 28.45 | -0.24 - 57.14 | 9.68 | 2.36 - 17.00 | 18.78 | 2.018 | 0.090 |
| 20 | 32.67 | 3.79 - 61.54 | 17.19 | 4.82 - 29.57 | 15.48 | 1.568 | 0.168 |
| 25 | 30.89 | 10.69 - 51.08 | 27.07 | 8.51 - 45.64 | 3.81 | 0.442 | 0.674 |
| 30 | 30.01 | 17.30 - 42.71 | 35.35 | 16.70 - 54.01 | -5.35 | 0.754 | 0.479 |
| 35 | 29.75 | 22.78 - 36.71 | 38.48 | 18.04 - 58.91 | -8.73 | 1.287 | 0.246 |
| 40 | 27.01 | 20.29 - 33.73 | 38.25 | 18.55 - 57.95 | -11.24 | 1.718 | 0.137 |
| 50 | 25.22 | 16.67 - 33.77 | 36.33 | 13.27 - 59.40 | -11.11 | 1.438 | 0.201 |
| 60 | 23.05 | 13.96 - 32.14 | 32.78 | 13.48 - 52.09 | -9.73 | 1.451 | 0.197 |
| 90 | 18.16 | 9.32 - 26.99 | 23.42 | 11.88 - 34.96 | -5.26 | 1.152 | 0.293 |
| 120 | 12.30 | 1.81 - 22.80 | 13.58 | 7.58 - 19.58 | -1.27 | 0.335 | 0.749 |
| 150 | 8.46 | -1.41 - 18.33 | 7.61 | 2.31 - 12.90 | 0.86 | 0.244 | 0.816 |
| 180 | 4.16 | -5.26 - 13.59 | 3.87 | -3.12 - 10.86 | 0.30 | 0.080 | 0.939 |
| 210 | 2.90 | -4.42 - 10.22 | 0.13 | -1.03 - 1.29 | 2.77 | 1.189 | 0.279 |

*Multiple Unpaired T test. 95% confidence interval (95% CI).

**Supplemental Table 4.** Mean delta insulin Humalog net AUC^#^ (mU^×^min/L) difference*

| Post injection (minutes) | Mean (glucagon group) | 95% CI | Mean (control group) | 95% CI | Difference | T ratio | P-value |
| --- | --- | --- | --- | --- | --- | --- | --- |
| 1 | 1.90 | -2.68 - 6.48 | 0.28 | -0.62 - 1.20 | 1.61 | 1.101 | 0.313 |
| 5 | 65.12 | -34.09 - 164.3 | 8.90 | -3.91 - 21.70 | 56.22 | 1.789 | 0.124 |
| 10 | 176.7 | -72.86 - 426.3 | 28.22 | -7.32 - 63.76 | 148.5 | 1.875 | 0.110 |
| 15 | 305.7 | -85.98 - 697.4 | 64.55 | -4.79 -134.0 | 241.2 | 1.930 | 0.102 |
| 20 | 458.5 | -73.15 - 990.2 | 131.7 | 13.54 - 250.0 | 326.8 | 1.910 | 0.105 |
| 25 | 617.4 | -34.76 - 1270 | 242.4 | 48.19 - 436.6 | 375.0 | 1.754 | 0.130 |
| 30 | 769.6 | 35.75 - 1503 | 398.4 | 113.2 - 683.7 | 371.2 | 1.500 | 0.184 |
| 35 | 919.0 | 137.1 - 1701 | 583.0 | 203.9 - 962.2 | 336.0 | 1.230 | 0.265 |
| 40 | 1061 | 251.6 - 1870 | 774.8 | 302.9 - 1247 | 286.1 | 0.972 | 0.369 |
| 50 | 1322 | 484.8 - 2159 | 1148 | 478.4 - 1817 | 174.3 | 0.518 | 0.623 |
| 60 | 1563 | 719.2 - 2408 | 1493 | 623.7 - 2363 | 70.07 | 0.184 | 0.860 |
| 90 | 2182 | 1276 - 3087 | 2336 | 1037 - 3635 | -154.9 | 0.311 | 0.766 |
| 120 | 2638 | 1583 - 3693 | 2891 | 1372 - 4410 | -252.9 | 0.435 | 0.678 |
| 150 | 2950 | 1698 - 4201 | 3209 | 1572 - 4846 | -259.2 | 0.400 | 0.703 |
| 180 | 3144 | 1724 - 4565 | 3381 | 1614 - 5148 | -236.7 | 0.332 | 0.751 |
| 210 | 3260 | 1691 - 4828 | 3444 | 1593 - 5295 | -184.0 | 0.241 | 0.817 |

*Multiple Unpaired T test. 95% confidence interval (95% CI).

^#^Area Under the Curve (AUC).

**GLUCOSE INFUSION RATE ANALYSES**

**Insulin Lyumjev^®^ and insulin Humalog^®^**

**
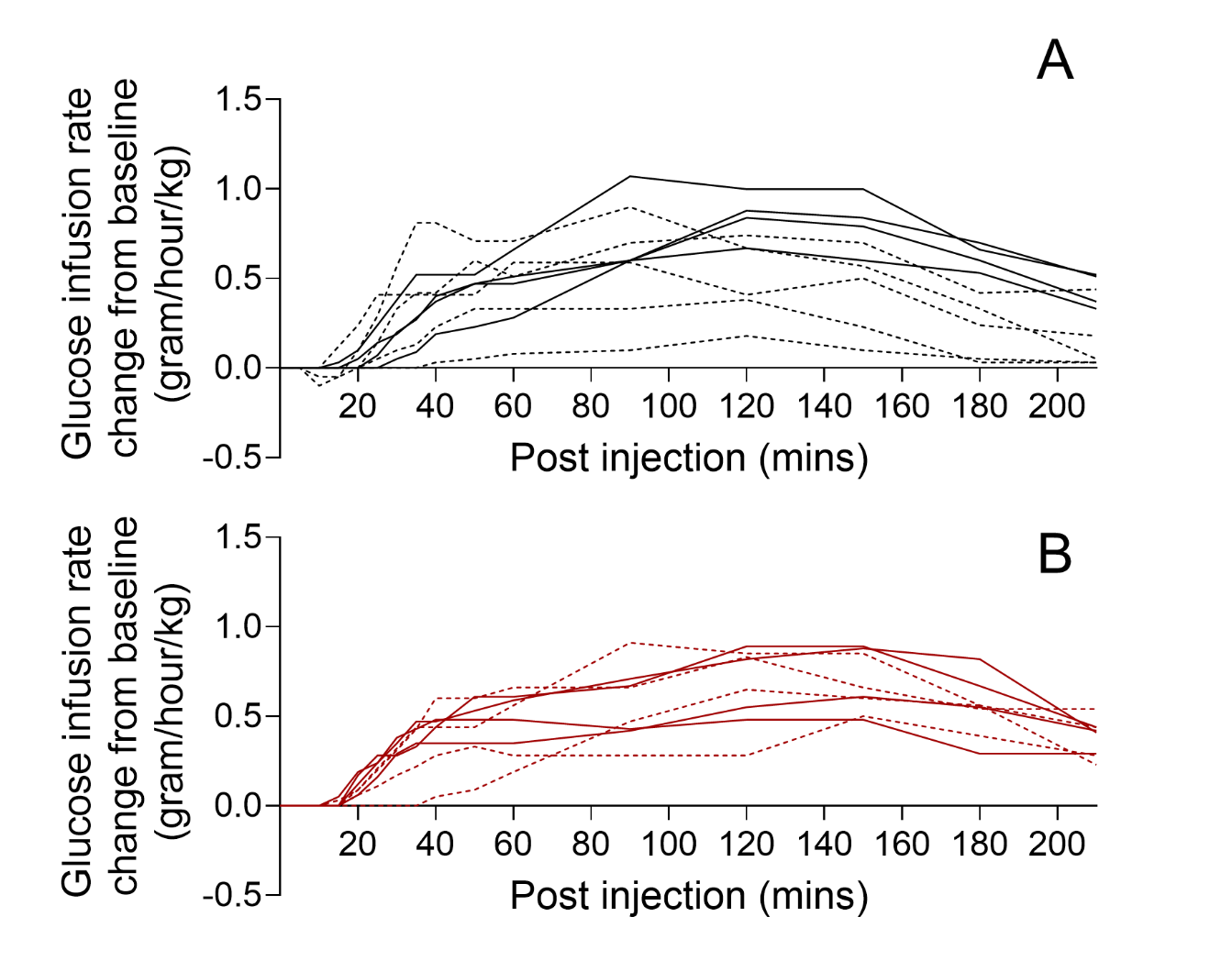
**

**Supplemental Figure 4**. Glucose infusion rate change from baseline after (A) insulin Lyumjev^®^ (Black dashed line) and premixed insulin Lyumjev^®^/glucagon (black line) and (B) Insulin Humalog^®^ (red dashed line) and premixed insulin Humalog^®^/glucagon (red line) injection.

**Supplemental Table 5**. Glucose infusion rate change from baseline (gram/hour/kg) difference after insulin Lyumjev^®^ injection *

| Post injection (minutes) | Mean (glucagon group) | 95% CI | Mean (control group) | 95% CI | Difference | T ratio | P-value |
| --- | --- | --- | --- | --- | --- | --- | --- |
| 1 | 0 | - | 0 | - | - |  | - |
| 5 | 0 | - | 0 | - | - |  | - |
| 10 | 0 | - | -0.03 | -0.09 - 0.03 | 0.03 | 1.323 | 0.227 |
| 15 | 0.01 | -0.03 - 0.05 | 0.05 | -0.08 - 0.09 | -0.04 | 0.230 | 0.215 |
| 20 | 0.13 | 0.04 - 0.23 | 0.10 | -0.06 - 0.20 | 0.03 | 1.131 | 0.755 |
| 25 | 0.16 | 0.15 - 0.31 | 0.17 | -0.03 - 0.39 | -0.01 | 0.584 | 0.878 |
| 30 | 0.22 | 0.25 - 0.40 | 0.28 | -0.01 - 0.57 | -0.06 | 0.361 | 0.640 |
| 35 | 0.30 | 0.29 - 0.50 | 0.36 | -0.03 - 0.74 | -0.06 | 0.254 | 0.752 |
| 40 | 0.33 | 0.34 - 0.53 | 0.38 | 0.02 - 0.74 | -0.05 | 0.370 | 0.795 |
| 50 | 0.39 | 0.32 - 0.67 | 0.43 | 0.10 - 0.74 | -0.04 | 0.524 | 0.840 |
| 60 | 0.41 | 0.32 - 0.70 | 0.44 | 0.14 - 0.75 | -0.03 | 0.469 | 0.840 |
| 90 | 0.46 | 0.31 - 0.80 | 0.53 | 0.13 - 0.91 | -0.07 | 0.194 | 0.731 |
| 120 | 0.54 | 0.37 - 1.00 | 0.48 | 0.19 - 0.76 | 0.06 | 1.437 | 0.733 |
| 150 | 0.55 | 0.39 - 1.04 | 0.42 | 0.11 - 0.73 | 0.13 | 1.913 | 0.505 |
| 180 | 0.41 | 0.23 - 0.94 | 0.22 | 0.00 - 0.43 | 0.20 | 2.807 | 0.253 |
| 210 | 0.27 | 0.28 - 0.50 | 0.15 | -0.07 - 0.36 | 0.12 | 2.595 | 0.344 |

*Multiple Unpaired T test. 95% confidence interval (95% CI).

**Supplemental Table 6**. Glucose infusion rate change from baseline (gram/hour/kg) difference after insulin Humalog^®^ injection *

| Post injection (minutes) | Mean (glucagon group) | 95% CI | Mean (control group) | 95% CI | Difference | P-value |
| --- | --- | --- | --- | --- | --- | --- |
| 1 | 0 | - | 0 | - | - | - |
| 5 | 0 | - | 0 | - | - | - |
| 10 | 0 | - | 0 | - | - | - |
| 15 | 0.01 | -0.02 - 0.03 | 0.01 | -0.02 - 0.03 | 0.00 | >0.999 |
| 20 | 0.06 | -0.01 - 0.13 | 0.04 | -0.04 - 0.11 | 0.02 | 0.508 |
| 25 | 0.13 | -0.02 - 0.29 | 0.11 | -0.05 - 0.28 | 0.02 | 0.813 |
| 30 | 0.20 | -0.04 - 0.44 | 0.21 | -0.01 - 0.42 | -0.01 | 0.962 |
| 35 | 0.27 | -0.06 - 0.60 | 0.29 | 0.01 - 0.57 | -0.02 | 0.902 |
| 40 | 0.34 | -0.03 - 0.72 | 0.37 | 0.15 - 0.59 | -0.03 | 0.846 |
| 50 | 0.37 | 0.02 - 0.71 | 0.42 | 0.21 - 0.63 | -0.06 | 0.663 |
| 60 | 0.42 | 0.07 - 0.78 | 0.48 | 0.23 - 0.73 | -0.06 | 0.688 |
| 90 | 0.58 | 0.15 - 1.01 | 0.72 | 0.34 - 1.09 | -0.14 | 0.471 |
| 120 | 0.65 | 0.23 - 1.07 | 0.85 | 0.63 - 1.07 | -0.20 | 0.237 |
| 150 | 0.65 | 0.42 - 0.89 | 0.81 | 0.54 - 1.07 | -0.16 | 0.210 |
| 180 | 0.51 | 0.38 - 0.64 | 0.62 | 0.50 - 0.74 | -0.11 | 0.094 |
| 210 | 0.37 | 0.14 - 0.60 | 0.43 | 0.28 - 0.59 | -0.06 | 0.513 |

*Multiple Unpaired T test. 95% confidence interval (95% CI).

**Supplemental Table 7**. Glucose infusion rate change from baseline (gram/hour/kg) difference in T_max_ and C_max_ after insulins Lyumjev^®^ and Humalog^®^ injection*

|  | T_max_  mean [± 95%CI] | C_max_  mean [± 95%CI] |
| --- | --- | --- |
| Lyumjev^®^/Glucagon^§^ (n=4), (gram/hour/kg) | 115.0 [32.32 - 197.68] | 0.72 [0.39 - 1.04] |
| Lyumjev^®^ control^#^ (n=5), (gram/hour/kg) | 102.0 [68.68 - 135.32] | 0.56 [0.20 - 0.91] |
| Difference | 13.0 | -0.17 |
| P-value | 0.640 | 0.387 |
| Humalog^®^/Glucagon^§^ (n=4), (gram/hour/kg) | 120.0 [81.02 - 158.98] | 0.72 [0.43 - 1.01] |
| Humalog^®^ control^#^ (n=4), (gram/hour/kg) | 112.5 [88.63 - 136.37] | 0.87 [0.60 - 1.13] |
| Difference | 7.50 | 0.143 |
| P value | 0.620 | 0.292 |

^#^Each pig received a 60 second SC infusion of 10 U of insulin.

^§^Insulin (10 U) was premixed with glucagon (55 ng).

T_max_, time till mean maximum concentration; C_max_, insulin maximum concentration (delta mean (95% confidence interval [95% CI]))

T_max_ and C_max_, obtained directly from real time data.


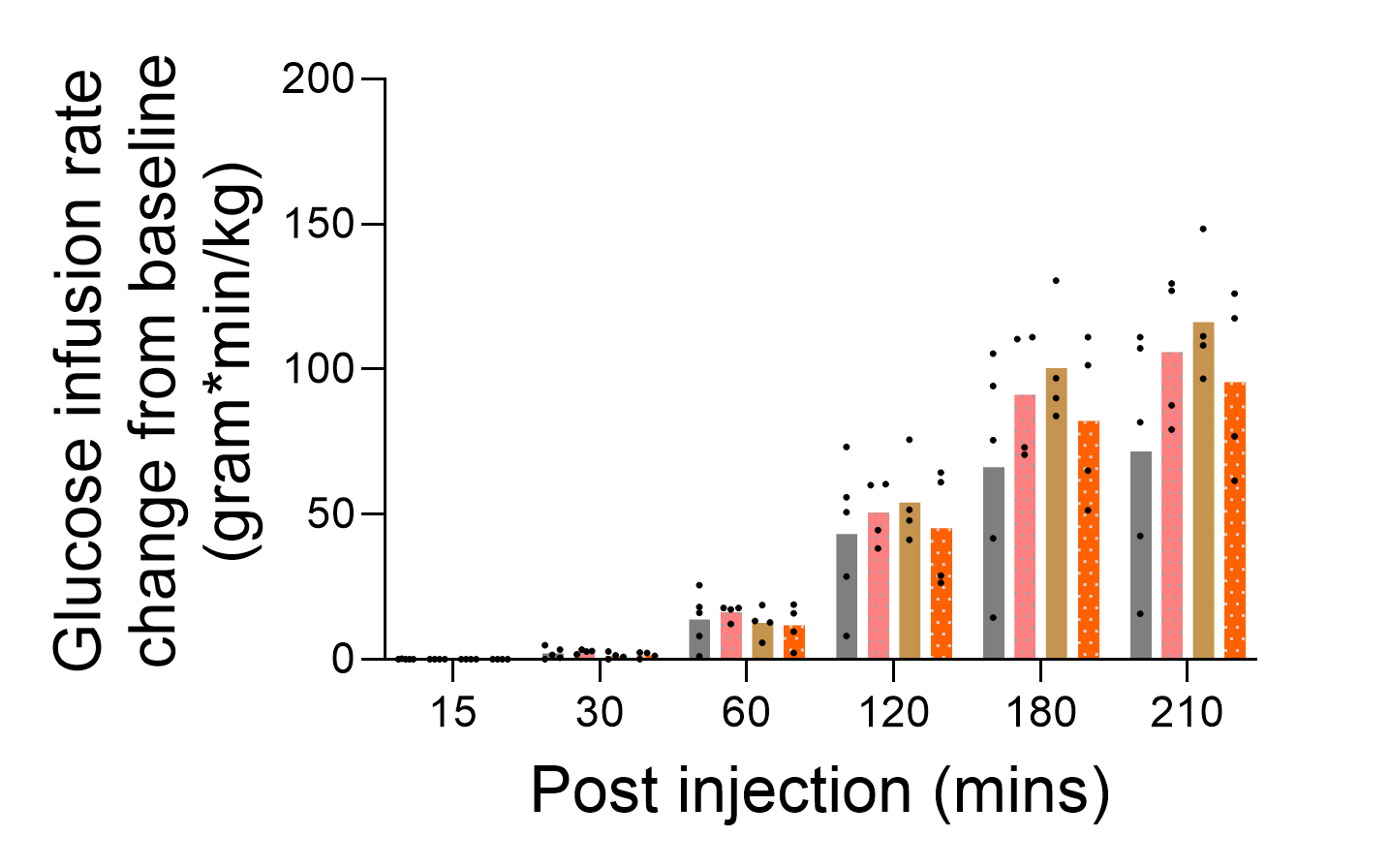


**Supplemental Figure 5**. Glucose infusion rate change from baseline, net Area Under the Curve (net AUC) summary for all series of experiments. Grey column, Control group (insulin Lyumjev); pink with dotes column, Glucagon group (pre-mixed insulin Lyumjev/Glucagon); Brown column, control group (insulin Humalog); orange with dotes column, glucagon group (pre-mixed insulin Humalog/Glucagon). Each dote on the columns represent individual pig.

**Supplemental Table 8.** Mean delta glucose infusion rate net AUC^#^ (gram^×^min/L) difference after insulin Lyumjev^®^ injection*

| Post injection (minutes) | Mean (glucagon group) | 95% CI | Mean (control group) | 95% CI | Difference | P-value |
| --- | --- | --- | --- | --- | --- | --- |
| 1 | 0 | - | 0 | - | - | - |
| 5 | 0 | - | 0 | - | - | - |
| 10 | 0 | - | 0 | - | - | - |
| 15 | 0.03 | -0.07 - 0.13 | 0.06 | -0.11 - 0.23 | -0.03 | 0.707 |
| 20 | 0.40 | 0.01 - 0.79 | 0.29 | -0.36 - 0.94 | 0.11 | 0.711 |
| 25 | 1.31 | 0.55 - 2.07 | 0.91 | -0.55 - 2.36 | 0.41 | 0.539 |
| 30 | 2.70 | 1.67 - 3.73 | 2.06 | -0.48 - 4.59 | 0.65 | 0.566 |
| 35 | 4.50 | 3.21 - 5.79 | 3.65 | -0.32 - 7.61 | 0.85 | 0.622 |
| 40 | 6.58 | 4.88 - 8.27 | 5.48 | -0.17 - 11.13 | 1.09 | 0.656 |
| 50 | 11.21 | 8.47 - 13.95 | 9.48 | 0.74 -1 8.22 | 1.73 | 0.649 |
| 60 | 16.21 | 11.91 - 20.51 | 13.80 | 2.09 - 25.51 | 2.41 | 0.639 |
| 90 | 32.19 | 22.09 - 42.29 | 28.32 | 6.31 - 50.33 | 3.87 | 0.693 |
| 120 | 50.83 | 33.06 - 68.59 | 43.32 | 11.83 - 74.81 | 7.51 | 0.602 |
| 150 | 71.83 | 45.06 - 98.59 | 56.76 | 17.17 - 96.35 | 15.07 | 0.425 |
| 180 | 91.29 | 55.42 - 127.2 | 66.27 | 19.45 - 113.1 | 25.02 | 0.283 |
| 210 | 105.9 | 64.21 - 147.5 | 71.67 | 20.10 - 123.2 | 34.21 | 0.197 |

*Multiple Unpaired T test. 95% confidence interval (95% CI).

^#^Area Under the Curve (AUC).

**Supplemental Table 9.** Mean delta glucose infusion rate net AUC^#^ (gram^×^min/L) difference after insulin Humalog^®^ injection*

| Post injection (minutes) | Mean (glucagon group) | 95% CI | Mean (control group) | 95% CI | Difference | P-value |
| --- | --- | --- | --- | --- | --- | --- |
| 1 | 0 | - | 0 | - | - | - |
| 5 | 0 | - | 0 | - | - | - |
| 10 | 0 | - | 0 | - | - | - |
| 15 | 0.02 | -0.04 - 0.08 | 0.02 | -0.04 - 0.08 | 0.00 | >0.999 |
| 20 | 0.19 | -0.06 - 0.44 | 0.13 | -0.17 - 0.43 | 0.06 | 0.662 |
| 25 | 0.66 | -0.13 - 1.45 | 0.51 | -0.38 - 1.39 | 0.16 | 0.690 |
| 30 | 1.49 | -0.29 - 3.26 | 1.30 | -0.50 - 3.10 | 0.19 | 0.821 |
| 35 | 2.67 | -0.53 - 5.87 | 2.54 | -0.49 - 5.56 | 0.13 | 0.927 |
| 40 | 4.21 | -0.71 - 9.12 | 4.19 | -0.05 - 8.43 | 0.02 | 0.993 |
| 50 | 7.74 | -0.66 - 16.15 | 8.15 | 1.92 - 14.38 | -0.41 | 0.906 |
| 60 | 11.68 | -0.11 - 23.48 | 12.66 | 4.23 - 21.09 | -0.98 | 0.837 |
| 90 | 26.72 | 4.96 - 48.48 | 30.63 | 13.77 - 47.48 | -3.91 | 0.667 |
| 120 | 45.21 | 12.86 - 77.55 | 54.10 | 30.14 - 78.06 | -8.89 | 0.508 |
| 150 | 64.78 | 23.86 - 105.7 | 78.93 | 49.67 - 108.2 | -14.14 | 0.405 |
| 180 | 82.26 | 36.86 - 127.7 | 100.4 | 67.16 - 133.6 | -18.12 | 0.345 |
| 210 | 95.53 | 45.85 - 145.2 | 116.2 | 80.65 - 151.8 | -20.67 | 0.323 |

*Multiple Unpaired T test. 95% confidence interval (95% CI).

^#^Area Under the Curve (AUC).

.

**Blood glucose level/euglycemic clamp**

**Insulin Lyumjev^®^ and insulin Humalog^®^**


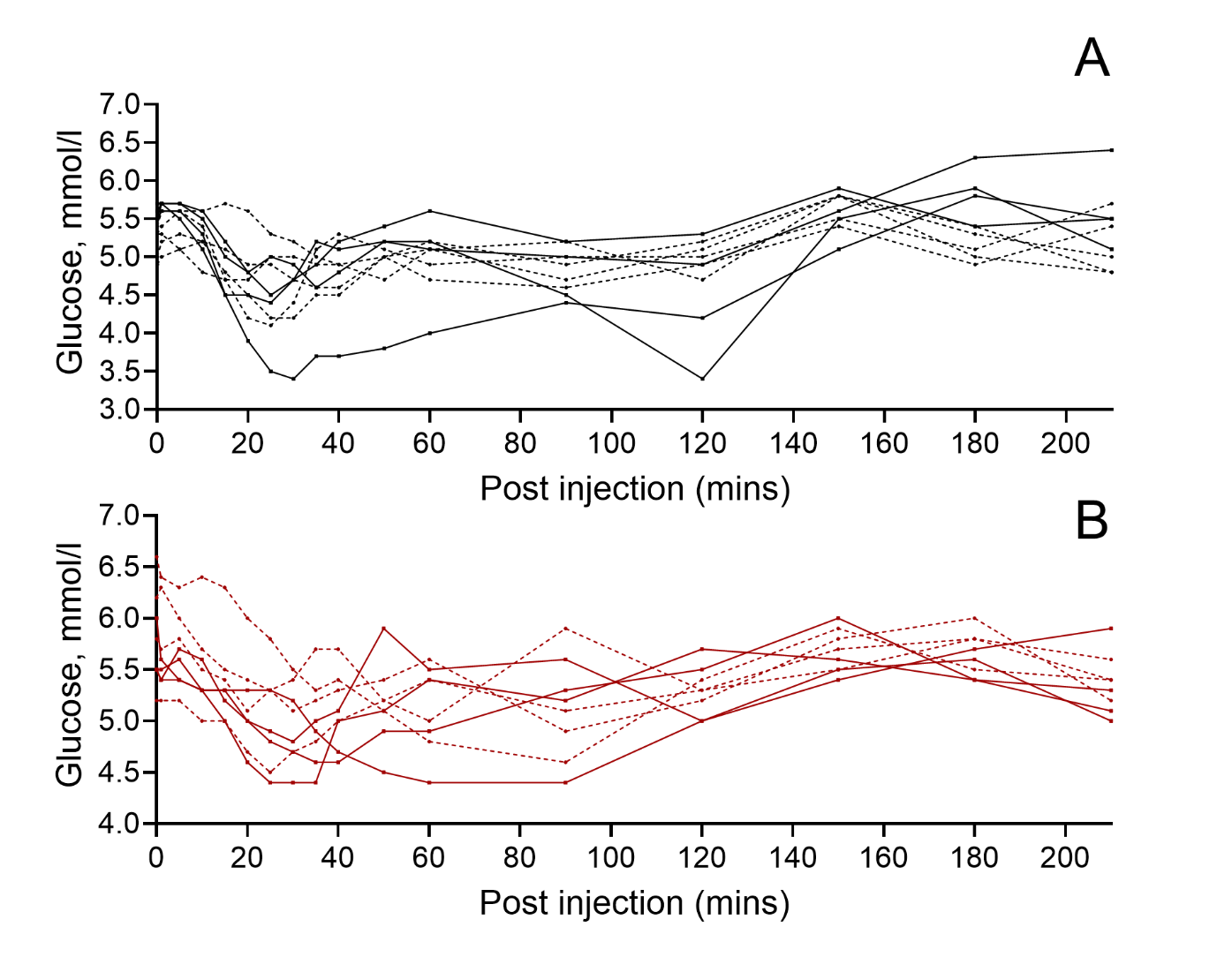


**Supplemental Figure 6.** Blood glucose level change from baseline after (A) insulin Lyumjev^®^ (Black dashed line) and premixed insulin Lyumjev^®^/glucagon (black line) during euglycemic clamp and (B) Insulin Humalog^®^ (red dashed line) and premixed insulin Humalog^®^/glucagon (red line) injection during euglycemic clamp.
